# Identification of Genetic Factors Underlying the Association between Sodium Intake Habits and Hypertension Risk

**DOI:** 10.3390/nu12092580

**Published:** 2020-08-25

**Authors:** Yu-Jin Kwon, Jung Oh Kim, Jae-Min Park, Ja-Eun Choi, Da-Hyun Park, Youhyun Song, Seong-Jin Kim, Ji-Won Lee, Kyung-Won Hong

**Affiliations:** 1 Department of Family Medicine, Yongin Severance Hospital, Yonsei University College of Medicine, 363, Dongbaekjukjeon-daero, Giheung-gu, Yongin-si 16995, Korea; digda3@yuhs.ac; 2 Theragen Bio Co., Ltd., Suwon 16229, Korea; jungoh.kim@therabio.kr (J.O.K.); jaeun.choi@therabio.kr (J.-E.C.); dahyun.park@therabio.kr (D.-H.P.); jasonsjkim@therabio.kr (S.-J.K.); 3 Department of Family Medicine, Gangnam Severance Hospital, Yonsei University College of Medicine, 211 Eonju-ro, Gangnam-gu, Seoul 06273, Korea; milkcandy@yuhs.ac (J.-M.P.); WLGMEO_O@yuhs.ac (Y.S.)

**Keywords:** sodium intake, hypertension, single-nucleotide polymorphism

## Abstract

The role of sodium in hypertension remains unresolved. Although genetic factors have a significant impact on high blood pressure, studies comparing genetic susceptibility between people with low and high sodium diets are lacking. We aimed to investigate the genetic variations related to hypertension according to sodium intake habits in a large Korean population-based study. Data for a total of 57,363 participants in the Korean Genome and Epidemiology Study Health Examination were analyzed. Sodium intake was measured by a semi-quantitative food frequency questionnaire. We classified participants according to sodium intake being less than or greater than 2 g/day. We used logistic regression to test single-marker variants for genetic association with a diagnosis of hypertension, adjusting for age, sex, body mass index, exercise, alcohol, smoking, potassium intake, principal components 1, and principal components 2. Significant associations were defined as *p* < 5 × 10^−8^. In participants whose sodium intake was greater than 2 g/day, chromosome 6 open reading frame 10 (C6orf10)-human leukocyte antigen (HLA)-DQB1 rs6913309, ring finger protein (RNF)213 rs112735431, glycosylphosphatidylinositol anchored molecule-like (GML)- cytochrome P450 family 11 subfamily B member 1(CYP11B1) rs3819496, myosin light chain 2 (MYL2)-cut like homeobox 2 (CUX2) rs12229654, and jagged1 (JAG1) rs1887320 were significantly associated with hypertension. In participants whose intake was less than 2 g/day, echinoderm microtubule-associated protein-like 6(EML6) rs67617923 was significantly associated with hypertension. Genetic susceptibility associated with hypertension differed according to sodium intake. Identifying gene variants that contribute to the dependence of hypertension on sodium intake status could make possible more individualized nutritional recommendations for preventing cardiovascular diseases.

## 1. Introduction

Sodium is the most important electrolyte for maintaining extracellular fluid volume and regulating cellular membrane potential [1]. The importance of dietary sodium in regulating blood pressure (BP) has received much attention in the past. Hypertension has been the most important global risk factor for all-cause mortality and for cardiovascular mortality [2]. Many studies have demonstrated the association of sodium consumption with hypertension and risk of cardiovascular diseases (CVD) [3,4,5]. Therefore, the World Health Organization (WHO) recommends sodium intake of less than 2 g/day to reduce BP and the risk of CVD [6].

Under normal physiological adaptation to sodium intake, the pressure natriuresis curve is regulated by the renin–angiotensin system and renal sympathetic nerve activity [7]. Increased sodium intake suppressed angiotensin II and led to pressure natriuresis cure shifting, which increased renal sodium excretion [7]. Both epithelial sodium transporter and aldosterone level are also involved in adapting the dietary sodium intake. In patients with salt-sensitive increased BP, enhanced sodium reabsorption, changes in pressure natriuresis curve, a suppressed renin–angiotensin system, and gene polymorphisms in voltage-dependent Ca^2+^ channels and sodium-bicarbonate cotransporter were noted [8,9]. Furthermore, the levels of natriuretic peptides could be affected by excessive sodium intake, decreased potassium and magnesium intake, and metabolic diseases such as obesity [10,11,12].

However, the relationship between sodium intake and BP remains unresolved. A meta-analysis of 13 prospective studies with 177,035 participants reported that high salt intake is associated with significantly increased risk of stroke and total CVD [3]. Another meta-analysis that included 22 trials in hypertensive patients and 12 trials in normotensive participants reported that a salt reduction of 4.4 g/day led to a mean systolic blood pressure (SBP) change of −4.18 mm Hg (95% confidence interval [CI] −5.18 to −3.18, I2 = 75%), and a diastolic blood pressure (DBP) change of −2.06 mm Hg (CI, −2.67 to −1.45, I2 = 68%) [4]. Conversely, several studies reported an inverse association between sodium intake and CVD. Stolarz-Skrzypek et al. [13] found that SBP, but not DBP, was significantly correlated with 24 h urinary sodium excretion; however, the incidence of hypertension did not increase, and CVD risk decreased with increasing sodium excretion tertiles. Interestingly, some studies have shown a J-shaped association between sodium intake and CVD. Martin et al. [14] showed that sodium excretion rates greater than 7 g/day or less than 3 g/day were associated with increased risk of all CV events as well as CVD mortality, compared to sodium excretion of 4 to 5.99 g/day, using the two-cohort data. The same author [15] reaffirmed that estimated sodium intake of 3 to 6 g/day was associated with a low risk of CVD among 101,945 persons in 17 countries. These conflicting findings are due not only to differences among studies but also to the complexity of traits of hypertension. Essential hypertension, with varying or unknown pathology, accounts for 95% of all hypertension cases [16]. High BP is known to result from interaction among multiple factors, including genetic susceptibility, obesity, aging, sedentary life style, alcohol consumption, high salt intake (especially in salt-sensitive persons), and low potassium intake [16]. Genetic elements were reported to make a 30–70% contribution to BP variation [17,18]. Under similar environmental conditions, some individuals develop hypertension and others do not.

Single-nucleotide polymorphisms (SNPs), single base substitutions within the deoxyribonucleic acid (DNA) sequence, are the most common type of human genetic variation [19]. Inter-individual genetic variation is an important determinant of human nutritional requirements [20]. However, studies comparing genetic susceptibility associated with hypertension between people with low versus high sodium diets have been limited. Identifying gene variants that contribute to the association of hypertension with sodium intake could contribute to better understanding of the pathophysiology of hypertension, and offer opportunities to determine optimal nutrition status for individuals.

Therefore, we aimed to investigate the genetic variations involved in the relationship between hypertension and sodium intake, in a large Korean population-based study.

## 2. Materials and Methods

### 2.1. Study Population

The Korean Genome and Epidemiology Study (KoGES) is a large cohort study to find genetic and environmental factors, and their interactions, in non-communicable diseases, with government funding [21]. KoGES Health Examination (KoGES_HEXA), one of the subset cohorts of KoGES, consists of community dwellers and participants, aged ≥ 40 years at baseline recruited from the national health examinee registry.

In the current study, we included the total 58,701 participants who participated in KoGES_HEXA. We excluded participants in KoGES_HEXA from the present study if values were missing for BP, body mass index (BMI), waist circumference (WC), heart rate (HR), alcohol, smoking, or exercise (*n* = 1338). A total of 57,363 participants were included in the current study. Hypertensive patients (*n* = 15,245) were defined as those with SBP ≥ 140 mm Hg, DBP ≥ 90 mm Hg, or a history of hypertension or taking antihypertensive medication. Controls (*n* = 42,114) were defined as those without hypertension or taking anti-hypertensive drug or cardiovascular diseases.

**Figure 1:**
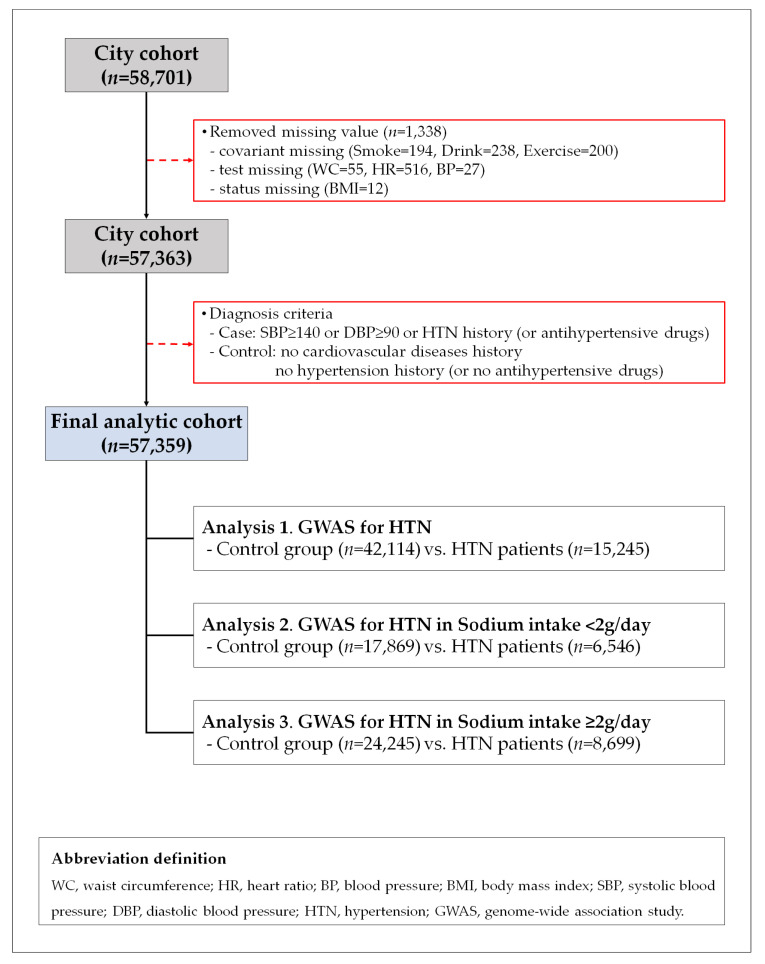
Flow chart of study population.

Figure 1 shows a flow chart describing this study. We treated the three analyses set. In analysis 1, we compared with control (*n* = 42,114) and hypertension patients (*n* = 15,245). In analysis 2, we compared with controls (*n* = 17,869) and hypertension patients (*n* = 6,546) in the participants with <2 g/day (*n* = 24,415). In analysis 3, we compared with controls (*n* = 24,245) and hypertension patients (*n* = 8699) in the participants with ≥2 g/day (*n* = 32,994). The study was approved by the institutional review board of Theragen Bio Co., Ltd. (approval number: 700062-20190819-GP-006-02).

### 2.2. Assessment of Dietary Sodium and Potassium Intake and Covariates

For dietary assessment, a semi-quantitative food frequency questionnaire (FFQ) involving 103 items was developed for the KoGES. Participants reported the frequency and amount of foods eaten over the past year. The results of the questionnaire were analyzed, with reference to a food composition database, to estimate intakes. FFQs are widely used as the primary dietary assessment tool in epidemiological studies [22]. We classified participants based on sodium intake, according to the WHO recommendation of 2 g/day [6]. The WC was measured midway between the bottom rib and the iliac crest. Body mass index (BMI) was calculated as weight (kg) divided by height (m) squared. Blood pressures in the seated position were measured twice, using a mercury sphygmomanometer. Smoking status was classified into three categories: non-smokers, ex-smokers and current smokers. Drinking status was classified into three groups: non-drinkers (those who drink alcohol fewer than 12 times a year, with one drink not exceeding one cup), ex-drinkers, and current drinkers. Exercise was defined as regular exercise sufficient to cause perspiration.

### 2.3. Genotyping

Fasting blood samples were collected into one serum separator tube and two ethylenediaminetetraacetic acid tubes. Blood DNA samples were prepared, and all samples were then transported to the National Biobank of Korea. The SNP genotypes of participants were extracted from the Korea Biobank array (referred to as KoreanChip), which was optimized for the Korean population and to demonstrate findings of genome-wide association study (GWAS) of blood biochemical traits. The KoreanChip comprised >833,000 markers, including >247,000 rare or functional variants, derived from sequencing data for over 2500 Koreans [23]. Detailed information about the KoreanChip was described in a previous study [23]. We applied the following criteria in the analysis of KoreanChip data, to control the quality of genotyping results: call rate >97%, minor allele frequency >0.01, missing genotype >0.01, Hardy-Weinberg equilibrium *p* > 0.000001. In addition, the genotype used in the analysis is genome data which imputed data from a dataset of 1000 genome phase 1 and 2 Asian panels.

### 2.4. Statistical Analysis

The data were presented either as mean ± standard deviation or as numbers (percentage). To compare participants with and without hypertension, we used two-tailed Student’s t-tests for continuous variables, or chi-squared tests for categorical variables. In addition, we performed principal component analysis (PCA) to reduce bias of genomic data according to the region where samples were collected, and used principal component (PC)1 and PC2 as covariates in statistical analyses. We used logistic regression to test single-marker variants for genetic association with a diagnosis of hypertension, while adjusting for age, sex, BMI, exercise, alcohol, smoking, potassium intake, PC1 and PC2. All statistical tests were based on an adjusted model using PLINK (ver. 1.07). *p* values < 5 × 10^−8^ were considered as statistically significant.

## 3. Results

### 3.1. General Characteristics of the Study Population

Table 1 shows the general characteristics of participant categorized according to sodium intake. There were 24,415 (42.6%) and 32,944 (57.4%) participants with sodium intakes <2 g/day or ≥2 g/day, respectively, and with respective mean ages of 54.1 and 53.6 years. The proportion of men was significantly higher among participants with sodium intake ≥2 g/day. SBP and DBP were also significantly higher in this group. The mean total cholesterol (TC) and low-density lipoprotein (LDL) cholesterol level were not different between two groups. The mean level of triglyceride (TG) was significantly higher in participants who intake sodium intake was ≥2 g/day, while the mean level of high-density lipoprotein (HDL) cholesterol was significantly lower in this group. The mean level of C-reactive protein (CRP) was not different between two groups. The proportions of exercise, drinking, and smoking were higher among participants with sodium intake ≥2 g/day. Sodium and potassium consumptions were significantly higher in participants with sodium intake ≥2 g/day (all *p* < 0.001). The sodium to potassium (Na/K) ratio was also significantly higher in participants with sodium intake ≥2 g/day (*p* < 0.001). Table 1 also presents characteristics of participants subcategorized according to the presence of hypertension (HTN). The mean age, BMI, WC, SBP, and DBP were significantly higher in hypertensive patients than controls, whether sodium intake was <2 or ≥2 g/day. The mean TC, HDL, LDL were significantly lower in hypertensive patients than controls, whether sodium intake was < or ≥2 g/day. The TG and CRP were significantly higher in hypertensive patients than controls, whether sodium intake was <2 or ≥2 g/day. Among participants with sodium intake <2 g/day, sodium and potassium intakes were lower for hypertensive patients (*p* = 0.038, and *p* < 0.001), whereas mean Na/K was higher in hypertensive patients than in controls (*p* < 0.001). Among participants with sodium intake ≥2 g/day, sodium intake was similar between hypertensives and controls (*p* = 0.433), while potassium intake was significantly lower (*p* < 0.001) and Na/K was higher in hypertensive patients (*p* < 0.001) than in controls.

### 3.2. SNPs Associated with Hypertension Based on Sodium Intake

Table 2 shows the SNPs most strongly associated or clustered with hypertension in the Korean subjects, according to their sodium intake. Odds ratios (OR) and 95% CIs were calculated using logistic regression analysis after adjusting for age, sex, BMI, alcohol consumption, smoking, physical activity, and potassium intake. SNPs rs16998073 and rs12509595 demonstrated significant association with hypertension risk both in participants with sodium intake <2 g/day and those with intake ≥2 g/day. SNPs rs1191582, rs11105378, and rs140473396 were significantly associated with a decreased risk of hypertension, both in participants with sodium intake <2 g/day and those with intake ≥ 2g/day. SNP rs67617923 was significantly associated with increased risk of hypertension only in participants with sodium intake <2 g/day (OR = 1.294 [1.187–1.410], *p* = 4.29 × 10^−9^). SNPs rs6913309 and rs112735431 were significantly associated with hypertension only in participants with sodium intake ≥2 g/day (OR = 1.145 [1.094–1.197], *p* = 4.23 × 10^−9^; and OR =1.706 [1.446–2.012], *p* = 2.38 × 10^−10^, respectively). SNPs rs3819496, rs12229654, and rs1887320 were significantly associated with decreased risk of hypertension in participants with sodium intake ≥2 g/day (OR = 0.892 [0.857–0.929], *p* = 3.73 × 10^−8^; OR = 0.834 [0.787–0.883], *p* = 5.25 × 10^−10^; and OR = 0.892 [0.859–0.925], *p* = 1.45 × 10^−9^, respectively). All SNPs that were found to be significantly related to hypertension are described in the Supplementary Tables (Table S1, SNPs significantly related to hypertension; Table S2, SNPs significantly related to hypertension in participants with sodium intake <2 g/day; Table S3, SNPs significantly related to hypertension in participants with sodium intake ≥2 g/day).

**Figure 2:**
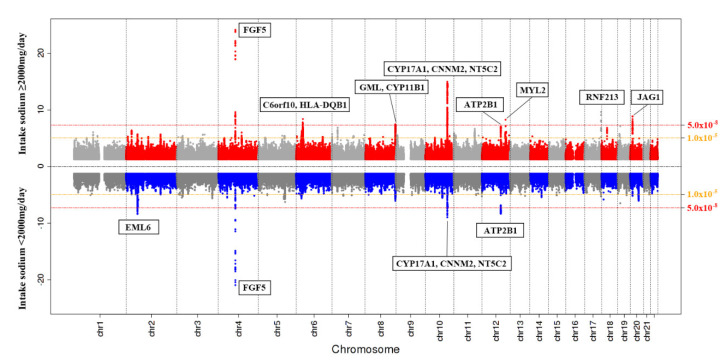
A Miami plot shows *p*-values for the SNP associations with hypertension in participants whose sodium intake was either <2 g/day or ≥2 g/day.

A Miami plot shows *p*-values for the SNP associations with hypertension in participants whose sodium intake was either <2 g/day or ≥2 g/day (Figure 2).

## 4. Discussion

This study identified both shared loci and sodium intake-specific loci related to hypertension. Fibroblast growth factor 5 (FGF5), PR domain zinc finger protein 8(PRDM8)-FGF5, 5’-nucleotidase, cytosolic II(NT5C2), ATPase plasma membrane Ca2+ transporting 1(ATP2B1), long intergenic non-protein coding RNA 936(LINC00936), and cyclin and CBS domain divalent metal cation transport mediator 2(CNNM2)-NT5C2 were commonly identified loci both in participants whose intakes were less than 2 g/day and in those with intakes greater than 2 g/day. Chromosome 6 open reading frame 10(C6orf10), human leukocyte antigen (HLA)-DQB1, ring finger protein (RNF)213, glycosylphosphatidylinositol anchored molecule-like (GML), cytochrome P450 family 11 subfamily B Member 1(CYP11B1), myosin light chain 2 (MYL2), cut like homeobox 2 (CUX2), and jagged1(JAG1) were significantly associated with hypertension in participants whose sodium intake was greater than 2 g/day, while loci in echinoderm microtubule-associated protein-like 6 (EML6) were significantly associated with hypertension in participants whose sodium intake was less than 2 g/day.

Guyton [24] established that long-term elevation of blood pressure is caused by vasoconstriction including the renal arteries or excess sodium retention through the kidney. The role of the kidney in BP control had been discovered by hypotension or hypertension caused by gene mutations which affect net renal sodium reabsorption [25]. For example, the loss of function mutations of the thiazide-sensitive NaCl symporter (e.g., Gitelman syndrome) impairs sodium reabsorption in the distal convoluted tubes and this results in a loss of sodium, potassium, and magnesium and a decrease in BP [25]. Enhanced tubular reabsorption of salt is important in the pathogenesis of obesity-related hypertension by regulating phosphorylation of Na^+^-K^+^-2Cl^−^ cotransporter and regulation of STE20/SPS1-related proline/alanine-rich kinase (SPAK)/oxidative-stress-responsive kinase-1 (OSR1) by AMP-activated protein kinase [26]. Recently, it was discovered that genetic variations at a number of loci increases susceptibility to hypertension in the context of environmental exposures through a variety of physiological mechanisms. Salt sensitivity has been more frequently observed in black people than white people and hypertensive persons than normotensive persons [27,28]. Therefore, races and individuals’ circumstance should be considered in salt intake and gene interaction studies [29,30]. In the current study, FGF5 rs16998073 and PRDM8-FGF5 rs12509595 were significantly associated with an increased risk of hypertension both in participants with sodium intake < 2 g/day and in those with intake ≥2 g/day. FGF5 rs16998073 was a well noted polymorphism in the largest GWAS performed by the Global Blood Pressure Genetics Consortium [31]. FGF5, a member of the fibroblast growth factor family, stimulates cell growth and proliferation of cardiac myocytes and promotes angiogenesis [32]. The association between FGF5 rs16998073 and hypertension was also recapitulated in a study of East Asians [33], and this polymorphism was shown to be associated with salt sensitivity in Koreans [34].

NT52C rs1191582, ATP2B1-LINC00936 rs11105378, and CNNM2-NT5C2 rs140473396 were significantly associated with decreased risk of hypertension, whether sodium intake was <2 or ≥2 g/day. NT52C rs11191582 is located in the gene-rich region near CYP17A1-CNNM2-NT5C2, which in GWAS was reported to contain a number of regulatory polymorphisms related to CVD [35,36]. Our study is the first to note the association of this polymorphism with hypertension. The association of ATP2B1 rs11105378 with hypertension was reported in European, Japanese and Korean studies [37]. ATP2B1 encodes the plasma membrane calcium transporting ATPase isoform 1, which plays a critical role in regulating blood pressure through alteration of intracellular calcium homeostasis and vasoconstriction in vascular smooth muscle cells [37,38]. CNNM2-NT5C2 rs140473396 was recently noted in a large, trans-ethnic study that included 776,078 participants from the Million Veteran Program, and in collaborating studies to identify the common variants, rare variants, and genetically predicted expression across multiple tissues of genes associated with blood pressure [39].

In the participants with sodium intake <2 g/day, we found significant association of rs67617923 in EML6 with increased risk of hypertension. While associations of several genetic variants in EML6 (e.g., rs17046380, rs72806698) with hypertension have been noted previously [40], rs67617923 is a novel genetic variant that was newly discovered in our study. Future studies to replicate this polymorphism association, and efforts to uncover the role of EML6 in blood pressure, are needed.

In participants with sodium intake ≥2 g/day, C6orf10-HLA-DQB1 rs6913309, and RNF213 rs112735431 were associated with increased risk of hypertension. C6orf10-HLA-DQB1 rs6913309 is another novel genetic variant that this study has newly discovered. An allele of HLA-DQB1 (which encodes a class II molecule expressed in antigen-presenting cells) increases the production of autoantibodies against angiotensin AT1 receptors, which was associated with essential hypertension in Chinese patients [41]. However, the exact role of HLA-DQB1 remains unclear. Lie et al. [42] found that the rs112735431 polymorphism of RNF213 was strongly associated with moyamoya disease in East Asian populations, including Chinese, Japanese, and Korean. This polymorphism has also been found to be related to intracranial artery steno-occlusive disease and moyamoya disease in Koreans [43]. The prevalence of moyamoya disease is 10 times higher in Japan and Korea than in Europe [44]. A previous study, which investigated the moyamoya disease susceptibility polymorphisms, reported that p.R4810K in RNF213 was found in the East Asian population but not in Southeast Asians [45]. Interestingly, the minor allele frequencies of the rs112735431 polymorphism were specified only in the East Asian population and in the present study. Although the physiologic function of RNF213 is not yet clear, previous studies found it to be involved in a novel signaling pathway in intracranial angiogenesis, and in the proliferation and maintenance of endothelial cells [42,46]. Ohkubo et al. [47] suggested that RNF213 promotes endothelial cell proliferation in response to inflammatory signals from the environment. Excess salt intake promotes vasoconstriction by decreasing nitric oxide production and increasing endothelial cell stiffness [48]. Furthermore, sodium intake is associated with systemic inflammation [49]. We may assume that excess sodium intake could be a provoking factor for the genetic effect of RNF213 on hypertension. Koizumi et al. [50] revealed that RNF213 was significantly associated with high BP in Japanese populations. Park et al. [43] also reported that the proportion of hypertension was higher in moyamoya diseases patients with the rs112735431 polymorphism of RNF213 than in those with wild type. This Korean GWAS was the first to note the association of this polymorphism with hypertension. Further studies to find association between the rs112735431 polymorphism in RNF213 and hypertension in other races/ethnicities are also needed.

We also found GML-CYP11B1 rs3819496, MYL2-CUX2 rs12229654, and JAG1 rs1887320 to be significantly associated with decreased risk of hypertension in participants with sodium intake ≥2 g/day. SNP rs3819496 represents a novel genetic variant, which was newly discovered in this study. Although MYL2-CUX2 rs12229654 and its association with hypertension were first reported in this study, a strong association of genetic variants of MYL2-CUX2 with high-density lipoprotein cholesterol was shown in a Korean GWAS meta-analysis, and it was replicated in a BioBank Japan GWAS, Health 2, and Shanghai Jiao Tong University cohort [51]. Another study conducted in Korea found that rs1229654 was also associated with dyslipidemia and diabetes [52]. Metabolic alteration due to rs1229654 might lead to the development of hypertension. Interestingly, the frequency of this polymorphism was determined only in the present study and in the East Asian population. Furthermore, persons carrying mutations in MYL-2, encoding slow cardiac myosin regulatory light chain 2, developed hypertrophic cardiomyopathy in the presence of hypertension or other risk factors for hypertrophy [53]. We may cautiously assume that excess salt intake might be an additional risk factor for hypertension or CVD in individuals with this genetic susceptibility. Association of JAG1 rs1887320 with hypertension and CVD risk was reported in Chinese cohorts [54,55].

Our study has certain limitations. We investigated the hypertension-related SNPs according to dietary sodium intake, as measured by FFQ. Although FFQ is a practical method to assess intake in large cohort studies, such questionnaires use a limited list of food items and cannot accurately consider additional salt intake via seasoning. Recall bias is another important limitation with FFQ. Second, we could not exclude the possibility of secondary hypertension due to a lack of information about it. Nevertheless, this is the first study to investigate hypertension-related SNPs according to sodium intake in a large population-based study. The current study identified previously well-reported SNPs related to hypertension. Furthermore, we identified several novel genetic variants associated with hypertension according to sodium intake.

## 5. Conclusions

In this large population-based study, we identified genetic susceptibility differences between participants whose sodium intake was less than 2 g/day and those whose intake was greater than 2 g/day. Discovering genetic predisposition for different sodium intakes would be helpful to establish the individualized medical nutrition therapy for disease management, and better targeted public health nutrition interventions. In further study, the effects and contributions of other confounding and interaction factors such as smoking, alcohol, and environmental factors on hypertension should be considered comprehensively.

## Figures and Tables

**Table 1:** General characteristics of study population based on sodium intake and according to prevalence of hypertension.

Characteristics	Sodium Intake		*p* ^1,†^	*p* ^2,†^	*p* ^3,†^	Sodium Intake <2 g/Day			Sodium Intake ≥2 g/Day		
	<2 g/Day (*n* = 24,415)	≥2 g/Day (*n* = 32,944)				Without HTN (*n* = 17,869)	With HTN (*n* = 6546)	*p* ^†^	Without HTN (*n* = 24,245)	With HTN (*n* = 8699)	*p* ^†^
Age (years,)	54.05 ± 7.90	53.58 ± 8.07	<0.0001	<0.0001	0.002	52.70 ± 7.70	57.73 ± 7.25	<0.0001	52.23 ± 7.85	57.36 ± 7.44	<0.0001
Sex (male, %)	7453 (30.5)	12,348 (37.5)	<0.0001 ^‡^	<0.0001 ^‡^	<0.0001 ^‡^	4929 (27.6)	2524 (38.6)	<0.0001 ^‡^	8264 (34.1)	4084 (46.9)	<0.0001 ^‡^
BMI (kg/m2)	24.02 ± 46.63	24.20 ± 34.68	0.606	0.743	<0.0001	23.29 ± 2.70	24.91 ± 2.92	<0.0001	23.59 ± 2.74	25.17 ± 2.95	<0.0001
WC (cm,)	80.08 ± 8.54	81.26 ± 8.65	<0.0001	<0.0001	<0.0001	78.72 ± 8.23	83.78 ± 8.29	<0.0001	79.93 ± 8.35	84.97 ± 8.40	<0.0001
SBP (mmHg)	122.14 ± 14.80	122.57 ± 14.73	0.001	<0.0001	0.042	117.57 ± 11.73	134.62 ± 15.12	<0.0001	118.07 ± 11.65	135.12 ± 15.14	<0.0001
DBP (mmHg)	75.44 ± 9.71	75.94 ± 9.72	<0.0001	<0.0001	0.001	72.89 ± 8.18	82.43 ± 10.11	<0.0001	73.42 ± 8.21	82.96 ± 10.15	<0.0001
HR (bpm)	69.30 ± 9.28	69.06 ± 9.00	0.002	0.012	0.09	68.94 ± 8.93	70.27 ± 10.11	<0.0001	68.73 ± 8.70	70.00 ± 9.74	<0.0001
TC (mg/dL)	197.19 ± 36.00	197.50 ± 35.45	0.307	0.424	0.558	197.88 ± 35.46	195.33 ± 37.35	<0.0001	198.16 ± 35.11	195.68 ± 36.30	<0.0001
TG (mg/dL)	122.06 ± 83.51	127.35 ± 87.03	<0.0001	<0.0001	0.0001	115.17 ± 77.32	140.86 ± 95.93	<0.0001	120.52 ± 82.17	146.35 ± 96.79	<0.0001
HDL-C (mg/dL)	54.23 ± 13.30	53.43 ± 13.04	<0.0001	<0.0001	<0.0001	55.05 ± 13.34	52.00 ± 12.91	<0.0001	54.23 ± 13.14	51.19 ± 12.49	<0.0001
LDL-C (mg/dL)	119.14 ± 32.36	119.40 ± 31.98	0.344	0.531	0.52	120.28 ± 31.72	116.00 ± 33.89	<0.0001	120.48 ± 31.46	116.36 ± 33.21	<0.0001
CRP (mg/dL)	0.14 ± 0.45	0.14 ± 0.34	0.333	0.432	0.639	0.13 ± 0.46	0.17 ± 0.41	<0.0001	0.13 ± 0.34	0.16 ± 0.34	<0.0001
Exercise status											
Yes (%)	13,103 (53.7)	18,250 (55.4)	<0.0001 ^‡^	<0.0001 ^‡^	0.441	9422 (52.7)	3681 (56.2)	<0.0001 ^‡^	13,304 (54.9)	4946 (56.9)	<0.0001 ^‡^
No (%)	11,312 (46.3)	14,694 (44.6)				8447 (47.3)	2865 (43.8)		10,941 (45.1)	3753 (43.1)	
Smoking status											
Non-smokers (%)	18,638 (76.3)	23,386 (71.0)	<0.0001 ^‡^	<0.0001 ^‡^	<0.0001 ^‡^	13,957 (78.1)	4681 (71.5)	<0.0001 ^‡^	17,779 (73.3)	5607 (64.5)	<0.0001 ^‡^
Ex-smokers (%)	3484 (14.3)	5586 (17.0)				2235 (12.5)	1249 (19.1)		3562 (14.7)	2024 (23.3)	
Current smokers (%)	2293 (9.4)	3972 (12.1)				1677 (9.4)	616 (9.4)		2904 (12.0)	1068 (12.3)	
Drinking status											
Non-drinker (%)	13,411 (54.9)	16,369 (49.7)	<0.0001 ^‡^	<0.0001 ^‡^	<0.0001 ^‡^	9880 (55.3)	3531 (53.9)	<0.0001 ^‡^	12,333 (50.9)	4036 (46.4)	<0.0001 ^‡^
Ex-drinker (%)	921 (3.8)	1212 (3.7)				605 (3.4)	316 (4.8)		767 (3.2)	445 (5.1)	
Current drinker (%)	10083 (41.3)	15363 (46.6)				7384 (41.3)	2699 (41.2)		11145 (46.0)	4218 (48.5)	
Total intake energy (kcal/day)	1498.32 ± 394.32	1923.57 ± 577.56	<0.0001	<0.0001	<0.0001	1502.56 ± 397.52	1486.77 ± 385.27	0.0056	1938.06 ± 588.37	1883.05 ± 544.22	<0.0001
Sugar (g/day)	275.09 ± 74.34	335.71 ± 92.28	<0.0001	<0.0001	<0.0001	274.83 ± 74.99	275.80 ± 72.54	0.3667	337.21 ± 93.79	331.51 ± 87.81	<0.0001
Fat (g/day)	21.03 ± 10.72	32.85 ± 19.94	<0.0001	<0.0001	<0.0001	21.54 ± 10.94	19.62 ± 9.96	<0.0001	33.57 ± 20.32	30.81 ± 18.71	<0.0001
Protein (g/day)	46.51 ± 14.39	68.25 ± 27.58	<0.0001	<0.0001	<0.0001	46.83 ± 14.50	45.64 ± 14.03	<0.0001	68.90 ± 28.03	66.45 ± 26.22	<0.0001
Sugar ratio	73.48 ± 6.45	70.41 ± 7.05	<0.0001	<0.0001	<0.0001	12.53 ± 2.23	12.32 ± 2.12	<0.0001	14.12 ± 2.55	14.02 ± 2.69	<0.0001
Fat ratio	5.58 ± 2.28	6.60 ± 2.40	<0.0001	<0.0001	<0.0001	5.71 ± 2.32	5.24 ± 2.12	<0.0001	6.70 ± 2.39	6.32 ± 2.40	<0.0001
Protein ratio	12.47 ± 2.20	14.09 ± 2.59	<0.0001	<0.0001	<0.0001	73.19 ± 6.57	74.28 ± 6.05	<0.0001	70.20 ± 7.01	71.01 ± 7.12	<0.0001
Na (mg/day)	1323.39 ± 435.98	3254.94 ± 1251.19	<0.0001	<0.0001	<0.0001	1328.30 ± 433.08	1310.05 ± 443.58	0.0038	3258.11 ± 1258.90	3245.85 ± 1229.30	0.4329
K (mg/day)	1591.80 ± 561.36	2694.00 ± 1046.87	<0.0001	<0.0001	<0.0001	1607.78 ± 564.81	1548.20 ± 549.57	<0.0001	2719.81 ± 1069.77	2621.77 ± 976.41	<0.0001
Na/K ratio	0.87 ± 0.28	1.26 ± 0.34	<0.0001	<0.0001	<0.0001	0.86 ± 0.28	0.88 ± 0.29	<0.0001	1.25 ± 0.34	1.29 ± 0.35	<0.0001

Data are presented either as mean ± standard deviation or as numbers (percentage). *p* values are calculated by two-tail Student’s *t*-test **^†^** or Chi-squared test ^‡^. ^1^ The *p*-value comparing the baseline characteristic between the sodium intake <2 g/day group and the sodium intake ≥2 g/day group, among all participants.^2^ The *p*-value comparing the baseline characteristic between the sodium intake <2 g/day group and sodium intake ≥2 g/day group, among participants without HTN. ^3^ The *p*-value comparing the baseline characteristic between the sodium intake <2 g/day group and sodium intake ≥2 g/day group, among participants with HTN. Sugar intake ratio = total sugar (gram) × 4 kcal/total energy intake (kcal) × 100; protein intake ratio = protein (gram) × 4 kcal/total energy intake (kcal) × 100; fat intake ratio (gram) × 9 kcal/total energy intake (kcal) × 100. BMI, body mass index; WC, waist circumference; SBP, systolic blood pressure; DBP, diastolic blood pressure; HR, heart rate; TC, total cholesterol; TG, triglyceride; HDL, high-density lipoprotein cholesterol; LDL, low density lipoprotein cholesterol; CRP, C-reactive protein. HTN, hypertension.

**Table 2:** Single-nucleotide polymorphisms (SNPs) most strongly associated with hypertension susceptibility loci in the Korean population, according to sodium intake.

SNP	Chr:BP	A1	MAF				Gene	Feature	Cluster SNP *	OR (95% CI)	*p*
			Present Study	EAS	EUR	AMR					
Participants with sodium intake <2 g/day											
rs67617923	2:54968517	A	0.063	0.074	0.160	0.098	*EML6*	intron variant	rs72806698; rs67246257; rs67514855	1.294 (1.187–1.410)	4.29 × 10^−9^
rs16998073	4:81184341	T	0.347	0.360	0.268	0.267	*FGF5*	upstream gene variant	rs12509595; rs10857147	1.245 (1.190–1.302)	1.14 × 10^−21^
rs11191582	10:104913653	A	0.227	0.265	0.089	0.193	*NT5C2*	intron variant	rs11191479; rs11191484; rs72050190; rs145010450; rs10883815	0.849 (0.806–0.895)	1.08 × 10^−9^
rs11105378	12:90090741	T	0.372	0.310	0.141	0.112	*ATP2B1–LINC00936*	intergenic region	rs2681485; rs7136259; rs11105377; rs1401982; rs1689040	0.874 (0.836–0.915)	4.67 × 10^−9^
Participants with sodium intake ≥2 g/day											
rs12509595	4:81182554	C	0.347	0.361	0.267	0.267	*PRDM8–FGF5*	intergenic region	rs16998073; rs10857147	1.228 (1.181–1.277)	7.46 × 10^−25^
rs6913309	6:32339840	A	0.212	0.139	0.310	0.244	*C6orf10–HLA-DQB1*	upstream gene variant	N/A	1.145 (1.094–1.197)	4.23 × 10^−9^
rs112735431	17:78358945	A	0.011	0.002	0.000	0.000	*RNF213*	missense variant	rs138309870	1.706 (1.446–2.012)	2.38 × 10^−10^
rs3819496	8:143923891	G	0.312	0.321	0.421	BP0.442	*GML–CYP11B1*	intron variant	rs3753123; rs143247792; rs4527848; rs4606038; rs28524031	0.892 (0.857.0.929)	3.73 × 10^−8^
rs140473396	10:104795885	GAC	0.247	0.285	0.097	0.197	*CNNM2–NT5C2*	intron variant	rs11191479; rs11191484; rs72050190; rs145010450; rs10883815	0.836 (0.800–0.873)	1.11 × 10^−15^
rs12229654	12:111414461	G	0.141	0.159	0.000	0.000	*MYL2–CUX2*	intergenic region	rs149607519; rs148177611; rs2188380; rs12227162	0.834 (0.787–0.883)	5.25 × 10^−10^
rs1887320	20:10965998	G	0.478	0.540	0.461	0.264	*JAG1*	intergenic region	rs6108787; rs1327235; rs6108789; rs913220	0.892 (0.859–0.925)	1.45x10^−9^

SNP, single-nucleotide polymorphism; Chr, chromosome; BP, base pair; EAS, East Asian; EUR, European; AMR, American; N/A, not applicable; MAF, major allele frequency; A1, minor allele; OR, odds ratio; 95% CI, 95% confidence interval. * The cluster SNP is the top five SNPs with an R^2^ value of 0.8 or higher, and within a ±200 kb range.

## Supplementary Materials

The following are available online at https://www.mdpi.com/2072-6643/12/9/2580/s1, Table S1: The hypertension susceptibility loci identified by GWAS signal in the Korean, Table S2: The significant association SNPs with hypertension susceptibility loci according to sodium intake (<2 g) in the Korean, Table S3: The significant association SNPs with hypertension susceptibility loci according to sodium intake greater than 2g per day in the Korean.
